# Next-Generation Sequencing Analysis of Gastric Cancer Identifies the Leukemia Inhibitory Factor Receptor as a Driving Factor in Gastric Cancer Progression and as a Predictor of Poor Prognosis

**DOI:** 10.3389/fonc.2022.939969

**Published:** 2022-06-30

**Authors:** Cristina Di Giorgio, Silvia Marchianò, Elisabetta Marino, Michele Biagioli, Rosalinda Roselli, Martina Bordoni, Rachele Bellini, Ginevra Urbani, Angela Zampella, Eleonora Distrutti, Annibale Donini, Luigina Graziosi, Stefano Fiorucci

**Affiliations:** ^1^ Department of Medicine and Surgery, University of Perugia, Perugia, Italy; ^2^ Azienda Ospedaliera Santa Maria della Misericordia, Perugia, Italy; ^3^ Department of Pharmacy, University of Naples Federico II, Naples, Italy

**Keywords:** gastric cancer (GC), metastases, peritoneal carcinomatosis (PC), trascriptome analysis, biomarker, LIF/LIFR axis, EC359

## Abstract

Gastric cancer (GC) is the third cause of cancer-related mortality worldwide. Nevertheless, because GC screening programs are not cost-effective, most patients receive diagnosis in the advanced stages, when surgical options are limited. Peritoneal dissemination occurs in approximately one-third of patients with GC at the diagnosis and is a strong predictor of poor outcome. Despite the clinical relevance, biological and molecular mechanisms underlying the development of peritoneal metastasis in GC remain poorly defined. Here, we report results of a high-throughput sequencing of transcriptome expression in paired samples of non-neoplastic and neoplastic gastric samples from 31 patients with GC with or without peritoneal carcinomatosis. The RNA-seq analysis led to the discovery of a group of highly upregulated or downregulated genes, including the leukemia inhibitory factor receptor (LIFR) and one cut domain family member 2 (ONECUT2) that were differentially modulated in patients with peritoneal disease in comparison with patients without peritoneal involvement. Both LIFR and ONECUT2 predicted survival at univariate statistical analysis. LIFR and its major ligand LIF belong to the interleukin-6 (IL-6) cytokine family and have a central role in immune system regulation, carcinogenesis, and dissemination in several human cancers. To confirm the mechanistic role of the LIF/LIFR pathway in promoting GC progression, GC cell lines were challenged *in vitro* with LIF and a LIFR inhibitor. Among several GC cell lines, MKN45 cells displayed the higher expression of the receptor, and their exposure to LIF promotes a concentration-dependent proliferation and epithelial–mesenchymal transition (EMT), as shown by modulation of relative expression of E-cadherin/vimentin along with JAK and STAT3 phosphorylation and acquisition of a migratory phenotype. Furthermore, exposure to LIF promoted the adhesion of MKN45 cells to the peritoneum in an *ex vivo* assay. These effects were reversed by the pharmacological blockade of LIFR signaling. Together, these data suggest that LIFR might have a major role in promoting disease progression and peritoneal dissemination in patients with GC and that development of LIF/LIFR inhibitors might have a role in the treatment of GC.

## Introduction

Gastric adenocarcinoma (GAC) is the fifth most common cancer but the third leading cause of cancer-related death (1–3) worldwide (1, 2), with a 5-year survival rate of ≈ 30% (3). The GC is a phenotypically and genotypically heterogeneous disease driven by multiple causative factors, including environmental factors and diet, Helicobacter (H.) pylori or Epstein–Barr virus (EBV) infection and host genetics (4, 5). According to the classical Lauren’s classification, GC is subdivided into three main histological subtypes: diffuse, intestinal, and mixed (6, 7). The diffuse subtype is generally more aggressive and predicts treatment resistance and poor prognosis (8). In contrast to the diffuse type, the intestinal GC is frequently associated with intestinal metaplasia, and H. pylori infection and its prevalence have faced a constant reduction in the last three decades in line with a progressive decrease of P. pylori infection in Western countries (9). Although the Lauren histological classification has been widely used over the past decades, its clinical significance remains limited because it does not reflect the molecular heterogeneity of the disease, which has been progressively elucidated by the diffuse application of next-generation sequencing (NGS) technologies to GC (10–13).

The Cancer Genome Atlas and the Asian Cancer Research Group have identified four distinct subtypes of GAC based on genetic and epigenetic signatures: EBV+, microsatellite instability, genome stability, and chromosomal instability (12, 14). These molecular patterns have been partially validated for clinical use, but there is still a need to better define negative or positive prognostic factors that will predict treatment efficacy.

Currently, radical chirurgical resection is the only therapeutic strategy that offers an effective cure for patients with GC (15). However, very often oncological curative surgery is prevented as most patients are diagnosed in an advanced stage with extensive lymph nodes involvement and distant metastases with limited survival rates. Thus, whereas Stage IIIC resected tumors are associated with 5-year survival rate of 18%, the survival rates for stage IA and IB tumors treated with surgery are 94% and 88%, respectively.

Metastasis is a multistep process (16, 17). A critical event in the formation of metastases is the epithelial–mesenchymal transition (EMT) process in which polarized epithelial cells undergo a process of de-differentiation, characterized by phenotypic changes that are supported by the profound reshaping of EMT biomarkers, including the downregulation of E-cadherin and the upregulation of N-cadherin or vimentin, along with the acquisition of migratory properties (18, 19) and a mesenchymal phenotype. Peritoneal metastases occur in approximately 30% of patients with GC at the time of diagnosis (20), and their presence impacts dramatically on patients survival (21). Furthermore, the peritoneal cavity is a common site of relapse of GC after treatment (22, 23). The poor response of peritoneal carcinomatosis (PC) to existing treatments highlights the need to better understand the promoting mechanisms and to identify molecular biomarkers that will predict development of PC in GC. Recently, NGS studies have shown that the leukemia inhibitory factor (LIF) is one of the highest expressed gene in various solid tumors, including stomach (24, 25), pancreas (26), colon (27), liver (28), and breast (29). Of relevance, LIF/LIF receptor (LIFR) overexpression in these tumors seems to predict a poor prognosis. LIF belongs to the interleukin-6 (IL-6) family of cytokines, promotes EMT, and is envisioned as a potential therapeutic target in many cancers (30). In target cells, LIF signaling is mediated by the formation of a heterodimeric complex assembled by the LIFRβ with the glycoprotein (GP) 130 subunit of IL-6 receptor. The GP130 subunit of the receptor is shared with other members of the IL-6 family of cytokines, whereas LIFRβ is shared only with oncostatin M, cardiotrophin-1, ciliary neurotrophic growth factor, and cardiotrophin-like cytokine. The downstream signaling of the LIF/LIFR pathway involves a JAK (Janus Kinase)-induced STAT3 (Signal Transducer And Activator Of Transcription 3) phosphorylation, AKT (Akt kinase), and mTor (mammalian target of rapamycin) (31–33). Furthermore, LIF is commonly upregulated in carboplatin and paclitaxel resistant cells, suggesting that LIF/LIFR overexpression might contribute to cancer chemoresistance (34). Nevertheless, the role of LIF in GC remains unclear, and some data suggest that LIF overexpression could be protective (35, 36).

In this paper, we report the transcriptome sequencing (RNA-seq) of paired samples of gastric mucosa and adenocarcinoma samples of patients with GC with or without PC and identified LIFR as one of the highest expressed genes in the GC. LIFR expression is a predictor of PC and poor prognosis. In addition, by using *in vitro* cancer cells and pharmacological approaches, we demonstrate that inhibition of LIF/LIFR signaling might have utility in the treatment of GC.

## Materials and Methods

### Patients and Specimens

Gastric carcinoma tissues were obtained from 31 patients undergoing surgical resection at the Department of Surgery at the Perugia University Hospital (Italy). Patients included in this series were from a larger cohort of patients with GC who underwent surgery for GC in the years 2014–2017. Patients were selected on the basis of availability of all clinical and histology data and at least 5-year follow-up in 2022, as well as paired tissue samples from normal and primary neoplastic tissues. None of them received chemotherapy or radiation before surgery. Specimen collection was freshly carried out during surgery by a biologist, and paired samples from of normal mucosa sample and neoplastic tissues were collected. Samples were transported to the Gastroenterology laboratory in RNA later and then snap-frozen at −80°C until use. Permission to collect post-surgical samples was granted to Prof. Stefano Fiorucci by the Ethics Committee of Umbria (CEAS), permit FI00001, no. 2266/2014 granted on February 19, 2014, and by University of Perugia Bioethics Committee, permit FIO0003, no. 36348 granted on May 6, 2020. An informed written consent was obtained by each patient before surgery.

### AmpliSeq Transcriptome

High-quality RNA was extracted from tumor gastric mucosa and healthy mucosa using the PureLink™ RNA Mini Kit (Thermo Fisher Scientific), according to the manufacturer’s instructions. RNA quality and quantity were assessed with the Qubit^®^ RNA HS Assay Kit and a Qubit 3.0 fluorometer followed by agarose gel electrophoresis. Libraries were generated using the Ion AmpliSeq™ Transcriptome Human Gene Expression Core Panel and Chef-Ready Kit (Thermo Fisher Scientific), according the manufacturer’s instructions. Briefly, 10 ng of RNA was reverse-transcribed with SuperScript™ Vilo™ cDNA Synthesis Kit (Thermo Fisher Scientific, Waltham, MA) before library preparation on the Ion Chef™ instrument (Thermo Fisher Scientific, Waltham, MA). The resulting cDNA was amplified to prepare barcoded libraries using the Ion Code™ PCR Plate, and the Ion AmpliSeq™ Transcriptome Mouse Gene Expression Core Panel (Thermo Fisher Scientific, Waltham, MA), Chef-Ready Kit, according to the manufacturer’s instructions. Barcoded libraries were combined to a final concentration of 100 pM and used to prepare Template-Positive Ion Sphere™ (Thermo Fisher Scientific, Waltham, MA) Particles to load on Ion 540™ Chips, using the Ion 540™ Kit-Chef (Thermo Fisher Scientific, Waltham, MA). Sequencing was performed on an Ion S5™ Sequencer with Torrent Suite™ Software v6 (Thermo Fisher Scientific). The analyses were performed with a range of fold change of <−2 and >+2 and a p-value of <0.05, using Transcriptome Analysis Console Software (version 4.0.2), certified for AmpliSeq analysis (Thermo-Fisher). The transcriptomic data have been deposited as dataset on Mendeley data repository (Mendeley Data, doi: 10.17632/9t86hd78sj.1).

### Gastric Cancer Cell Lines

Human gastric cell lines MKN74, MKN45, and KATO III were from the Japanase Collection of Research Bioresources, Human Science Resources Bank (Osaka, Japan). These cells were grown in RPMI 1640 (Sigma-Merk LIFe Science S.r.l. Milan, Italy) medium supplemented with 10% fetal bovine serum (FBS), 1% l-glutamine, 1% penicillin/streptomycin, in a humidified 5% CO_2_ atmosphere, 37°C. Cells, free from Mycoplasma contamination, confirmed by the use of Mycoplasma PCR Detection (Sigma) were regularly passaged to maintain exponential growth and used from early passages (<10 passages after thawing). To perform all experiments, cells were plated, serum-starved for 24 h, and stimulated for 8, 24, and 48 h.

### Real-Time PCR

The RNA was extracted from patient biopsies using the Trizol reagent (Invitrogen) and from cell lines using and Direct-zol™ RNA MiniPrep w/Zymo-Spin™ IIC Columns (Zymo Research, Irvine, CA, USA), according to the manufacturer’s protocol. After purification from genomic DNA by DNase I treatment (ThermoFisher Scientific, Waltham, MA USA), 2 µg of RNA from each sample was reverse-transcribed using the FastGene Scriptase Basic Kit (Nippon Genetics, Mariaweilerstraße, Düren, Germania) in a 20-μl reaction volume. Finally, 50 ng of cDNA was amp LIFied in a 20-μl solution containing 200 nM of each primer and 10 μl of the SYBR Select Master Mix (ThermoFisher Scientific). All reactions were performed in triplicate, and the thermal cycling conditions were as follows: 3 min at 95°C, followed by 40 cycles of 95°C for 15 s, 56°C for 20 s, and 72°C for 30 s, using a Step One Plus machine (Applied Biosystem). The relative mRNA expression was calculated accordingly to the 2^−ΔCt^ method. Primers used in this study were designed using the PRIMER3 (http://frodo.wi.mit.edu/primer3/) software using the NCBI (National Center for Biotechnology Information) database. RT-PCR (Reverse transcriptase-polymerase chain reaction) primers used in this study for human sample and human cell lines were as follows [forward (for) and reverse (rev)]: Cmyc (for: TCGGATTCTCTGCTCTCCTC; rev: TTTTCCACAGAAACAACATCG), E-cadherin (for: GAATGACAACAAGCCCGAAT; rev: TGAGGATGGTGTAAGCGATG), Snail1 (for: ACCCACACTGGCGAGAAG; rev: TGACATCTGAGTGGGTCTGG), and vimentin (for: TCAGAGAGAGGAAGCCGAAA; rev: ATTCCACTTTGCGTTCAAGG).

### Immunohistochemistry and Immunocytochemistry

Immunohistochemistry (IHC) was performed on paraffin-embedded human stomach. In brief, Ag retrieval was achieved by incubation of the slides for 90 min in the hot (95°C) sodium citrate buffer (pH 6.0) and 30 min of cooling at room temperature. Immunostaining technique was carried out using the commercial kit Elabscience^®^2-step plus Poly-HRP Anti-Rabbit/Mouse IgG Detection System (with DAB Solution) (Houston, TX 77079, USA.) Anti-LIFR Rabbit Polyclonal Antibody (Ab) (ab235908; Abcam, Cambridge, UK) was incubated overnight at 4°C. Subsequently, sections were incubated with Polyperoxidase anti-Mouse/Rabbit IgG and then with DAB Working Solution, both supplied by the kit. Slides were counterstained with hematoxylin, dehydrated through ethanol and xylene, and coverslipped using a xylene-based mounting medium.

Slides were observed under microscope and the photos were obtained with the Nikon DS-Ri2 camera, with magnification of ×20, ×40, and ×100. Immunocytochemistry (ICC) was performed on MKN45, untreated or treated with LIF (10 ng/ml; 14890-H02H, SinoBiological, Düsseldorfer, 65760 Eschborn, Germany). Cells were plate on slides using cytospined. The spots obtained were fixed in 4% formalin for 20 min and then submitted at the same procedure of immunostaining with the commercial kit Elabscience^®^2-step plus Poly-HRP Anti-Rabbit/Mouse IgG Detection System (with DAB Solution) (Houston, TX 77079, USA). After incubation with LIFR primary Ab and secondary Ab supplied by the kit, cells were counterstained with hematoxylin and then observed under microscope with magnification of ×100.

### Cell Proliferation Assay

The cell viability assay was done using the CellTiter 96 Aqueous One Solution Cell Proliferation Assay (Promega, Milano, Italy), a colorimetric method for accessing the number of viable cells in proliferation. The MTS assay protocol is based on the reduction of the MTS [3-(4,5-dimethylthiazol-2-yl)-5-(3-carboxymethoxyphenyl)-2-(4-sulfophenyl)-2H-tetrazolium] tetrazolium by cells into a colored formazan product that is soluble in cell culture media. Briefly, on day 0, MKN45 cells were seeded in RPMI 1640 complete medium at 36 × 10^3^ cells/100-µl well into 96-well tissue culture plate. On day 1, cells were serum-starved for 24 h, and on day 3, cells were primed with the LIFR major ligand, LIF (0.5, 5, 10, 50, and 100 ng/ml), or only with vehicle. In another experimental setting, on day 3, cells were triggered with LIF (10 ng/ml) plus LIFR antagonist, EC359 (25, 50, 100, and 1,000 nM) (MedChemExpress, NJ 08852, USA), and cell proliferation was assessed as mentioned above. Absorbance was measured using a 96-well reader spectrophotometer (490 nm). In these experiments, each experimental setting was replicated 10 folds. For analysis, the well background readings with the medium alone were subtracted from the samples readouts.

### Flow Cytometry

The intracellular flow cytometry staining for Ki-67 was performed using the following reagents: Ki-67 Monoclonal Antibody (SolA15), Alexa Fluor™ 488 (eBioscience™, San Diego, CA, United States), and DAPI (4',6-diamidin-2-fenilindolo) to characterize the cell cycle phases G0-G1, S-G2-M, and the apoptosis rate. Briefly, MK45 cells were seeded in six-well tissue culture plate (cell density 700 × 10^3^ per well) in 100 µl of RPMI 1640 medium supplemented with 10% FBS, 1% l-glutamine, and 1% penicillin and streptomycin at 37°C and 5% CO_2_. Cells were serum-starved for 24 h and then incubated with LIF (10 and 50 ng/ml) or vehicle for 48 h. In another experimental setting, cells were first challenged with LIF (10 ng/ml) alone or in combination with LIFR antagonists EC359 25 nM. Before intracellular IC-FACS (Immun cells-Fluorescence-Activated Cell Sorting) staining cells were fixed for 30 min in the dark using IC Fixation Buffer (eBioscience™) and then permeabilized using Permeabilization Buffer (10X) (eBioscience™). Flow cytometry analyses were carried out using a three-laser standard configuration ATTUNE NxT (LIFe Technologies, Carlsbad, CA). Data were analyzed using FlowJo software (TreeStar) and the gates set using a fluorescence minus one (FMO) control strategy. FMO controls are samples that include all conjugated Abs present in the test samples except one. The channel in which the conjugated Ab is missing is the one for which the FMO provides a gating control.

### Western Blot Analysis

MK45 cells were seeded in six-well tissue culture plate (cell density, 400 × 10^3^ per well) in 100 µl of RPMI 1640 medium supplemented with 10% FBS, 1% l-glutamine, and 1% penicillin and streptomycin at 37°C and 5% CO_2_. Cells were serum-starved for 24 h and then incubated with LIF (10 ng/ml) and EC359 (25 and 100 nM), alone or in combination, for 48 h. Total lysates were prepared by homogenization of MKN45 cells in Ripa buffer containing phosphatase and protease inhibitors. Protein extracts were electrophoresed on 12% acrylamide Tris-Glycine gel (Invitrogen), blotted to nitrocellulose membrane, and then incubated overnight with primary Abs against Jak1 (1:500; sc-7228, Santa Cruz Biotechnology), phospho-Jak1 (1:1,000; GTX25493, GeneTex), STAT3 (1:500; sc-8019, Santa Cruz Biotechnology), phosho-Stat3 (1:1,000; GTX118000, GeneTex), and Gapdh (1:1,000; bs2188R, Bioss Antibodies). Primary Abs were detected with the HRP (horseradish peroxidase)-labeled secondary Abs. Proteins were visualized by Immobilon Western Chemiluminescent Reagent (MilliporeSigma) and iBright Imaging Systems (Invitrogen). Quantitative densitometry analysis was performed using ImageJ software. The degree of JAK1 and STAT3 phosphorylation was calculated as the ratio between the densitometry readings of p-Jak1/Jak1 and p-STAT3/STAT3, respectively.

### Wound Healing Assay

MKN45 cells were seeded in RPMI 1640 complete medium at 800 × 10^3^ cells per well into 24-well plate and used at 70%–80% confluence rate (37). On the day 1, the cell monolayers were gently scraped vertically with a new 0.2-ml pipette tip across the center of the well; during the scratch, the medium was not removed to avoid cell death. After scratching, the well was gently washed twice with PBS (Phosphate buffered saline) (Euroclone, Milan, Italy) to remove the detached cells and cell debris, and finally, fresh medium containing LIF (10 ng/ml) and EC359 (100 nM), alone or in combination, was added into each well. Immediately after scratch creation, the 24-well plate was placed under a phase-contrast microscope, and the first image of the scratch was acquired (T = 0 h) using an OPTIKAM Pro Cool 5 – 4083.CL5 camera. Cells were grown for additional 48 h, and images were taken at 24 and 48 h. The gap distance between scarps borders was quantified by assessing that area between the two margins of the scratchs. All experiments were performed in triplicate.

### Cell Adhesion to Peritoneum

For these experiments, MKN45 cells were grown in a complete RPMI medium and on day 2, starved, and left untreated or incubated with LIF (10 ng/ml) and EC359 (100 nM), alone or in combination for 48 h. On day 5, mouse parietal peritoneum sections (~1.6 cm^2^) were placed in a 24-well culture plate, which had been filled with 1.0 ml of RPMI 1640 medium supplemented with 5% of FBS (38) and incubated with MKN45 cells. For this purpose, GC cells were first detached, fluorescently labeled with BCECF-AM (2',7'-bis-(2-carboxyethyl)-5-(and-6)-carboxyfluorescein) (10 μM) at 37°C for 30 min, and washed twice with PBS and after trypan blue staining; a suspension of living cells (5 × 10^5^ cells/ml in RPMI 1640) were seeded on the peritoneum in a 24-well plate; and the plate was incubated at 37°C for 60 min. After a gentle washing with PBS, the cells adherent to the peritoneum were lysed with 1.0 ml of Tris (50 mM) plus 1% SDS (Sodium dodecyl sulfate). Fluorescence intensity was measured with a fluorescence spectrophotometer (Ex = 490 nm and Em = 520 nm). Experiments were carried out in quintuplicate.

### Statistical Analysis

Patients’ descriptive analysis was generated, and their differences were investigated using Student’s t-test for quantitative data; normality test according to D’Agostino-Pearson was performed, and when not passed, quantitative data were compared using the Mann–Whitney test. For qualitative data, we used either the Fisher’s exact test or the Chi-square test. Overall survival analyses were carried out with the Kaplan–Meier method, and differences were evaluated using log-rank test. Only variables that achieved statistical significance in the univariate analysis were subsequently evaluated in the multivariate analysis using Cox’s proportional hazard regression model. ROC (receiver operating characteristic curve and Area Under the Curve) curves and AUC have also been calculated with the help of statistical software. A p-value of less than 0.05 was considered statistically significant. All statistical analyses were performed using the MedCalc Statistical Software version 14.8.1 (MedCalc Software, Ostend, Belgium), Prism 7.2 GraphPad, and SPSS, IBM version 23.

*In vitro* statistical analysis was carried out using the ANOVA followed by the nonparametric Mann–Whitney U-test or a two-tailed unpaired Student’s t-test comparisons (* p < 0.05) using the Prism 6.0 software (GraphPad San Diego, CA, USA).

## Results

### Patients

**Figure 1:**
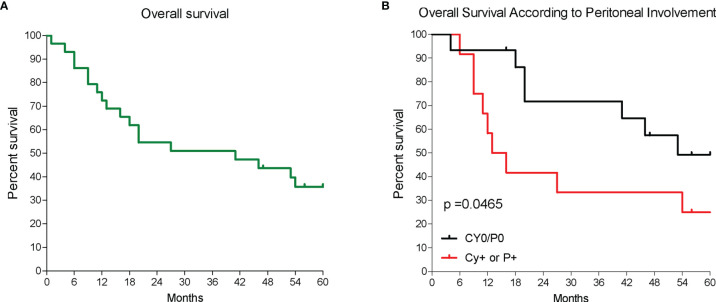
Patients survival. **(A)** Overall survival of a cohort of patients with GC and **(B)** overall survival of 31 patients according to the presence of peritoneal disease either macroscopically or microscopically; p < 0.05.

This study includes RNA-seq analysis of paired gastric samples from 31 patients with GC undergoing surgery at the Perugia University Hospital (2013–2019). Peritoneal metastasis dissemination was verified at surgery either macroscopically (P+) or microscopically (Cy+). This led to the identification of 19 patients with no peritoneal involvement (P0 and Cy0) and 12 who had peritoneal involvement (P+ or Cy+) at surgery. **Table 1** shows demographic characteristics, primary tumor features and surgical approaches followed in these patients. Patients were then followed up to 5 years after surgery, and, as shown in **Figure 1**, median survival time was 41 months and the 5-year overall survival rate was 35.7%. As shown in **Figure 1**, patients with peritoneal involvement have a significantly worse prognosis, whereas patients without peritoneal involvement had a median survival of 53 months (5-year survival rate, 49.2%), and the median survival time was 14.5 months in patients with PC (5-year survival rate, 25%).

**Table 1:** Clinical and pathological characterization of patients population at baseline.

Clinical pathological characteristics	Cy0 and P0 (n = 19)	Cy+ or P+ (n = 13)	P
Age*	76.3 ± 5.3	71± 12.7	N.S
**Gender**			
**Male**	10 (52.6%)	8 (61.5%)	N.S
**Female**	9 (47.4%)	5 (38.5%)	
N/L^**^	2.7 (1.2-22)	29 (1.6-5.7)	N.S
P/L**	136.4 (58.11-342.9)	144.1 (89.71-255-9)	N.S
L/M**	2.45 (0.97-6.68)	2.92 (1.14.03)	N.S
Surgery***			
**Subtotal**			
**Gastrectomy GaGastrectomy**	7 (36.9%)	8 (61.5%)	N.S
**Total**			
**Gastrectomy**	11 (57.9%)	5 (38.5%)	
**Lymphoadenectomy**			
Level***:			
**D1**	2 (10.5%)	2 (15.5%)	N.S
**D2**	11 (57.9%)	9 (69,2%)	
**D2+**	6 (31.6%)	1 (7,7%)	
Lauren Hystotype***:			
**Intestinal**	13 (68.4%)	6 (46.2%)	N.S
**Diffuse**	4 (21.1%)	6 (46,2%)	
**Mixed**	1 (5.3%)	1 (7.6%)	
**Signet Ring Cell:**			
**Yes**	1 (5.3%)	2 (15.4%)	N.S
**No**	18 (94.7%)	11 (84.6%)	
**pT**			
**2**	3 (15.8%)	1 (7.6%)	
**3**	9 (47.4%)	3 (23.1%)	N.S
**4a**	5 (26.3%)	7 (53.8%)	
**4b**	2 (10.5%)	2 (15.5%)	
**pN**			
**0**	5 (26.3%)	1 (7.6%)	
**1**	1 (5.3%)	1 (7.6%)	
**2**	3 (15.8%)	4 (30.8%)	N.S
**3a**	5 (26.3%)	2 (15.5%)	
**3b**	5 (26.3%)	5 (38.5%)	
**Stage**			
**I**	1 (5.4%)	0	
**II**	4 (21.0%)	0	
**IIIa**	4 (21.0%)	0	< 0.0001
**IIIb**	6(31.6)	0	
**IIIc**	4 (21.0%)	0	
**IV**	0	13 (100%)	
**Lymphonodal Harvasted**	46 (8-126)	34 (15-44)	0.05
**Ln ratio**	0.18 (0-0.85)	0.20 (0-0.75)	n.s
**Veno-Lymp. Invasion**			
**Yes**	17 (89.5%)	11 (84.6%)	
**No**	2 (10.5%)	1 (76%)	n.s
**Periner. Invasion**			
**Yes**	13 (68.4%)	10 ( 76.9%)	n.s
**No**	2 (10.5%)	3 (23.1%)	

Patients were subdivided according to the presence or not of peritoneal disease (Cyoand Povs. Cy+ or P+). * Mean and SD; **Median values and range; ***data of Lauren classification were missed in one patient from each group. ns, not statistic.

### Gene Expression Profile

**Figure 2:**
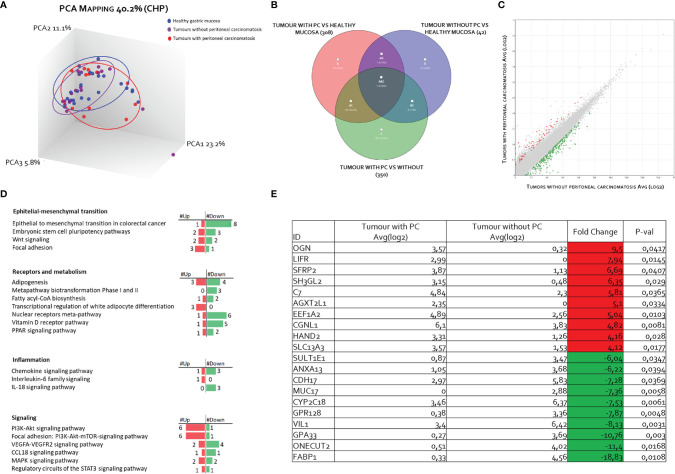
Transcriptome analysis of gastric cancer and paired normal tissues in 31 patients with advanced gastric cancer. **(A)** Heterogeneity characterization of gastric samples showed by principal component analysis (PCA) plot. **(B)** Venn diagram of differentially expressed genes showing the overlapping regions between the three comparison groups: gastric cancer with peritoneal carcinomatosis vs. healthy mucosa (red subset), gastric cancer without peritoneal carcinomatosis vs. healthy mucosa (blue subset), and gastric cancer with peritoneal carcinomatosis vs. gastric cancer without peritoneal carcinomatosis (green subset). **(C)** Scatter plots of transcripts differentially expressed between gastric cancer tissues with peritoneal carcinomatosis and gastric cancer tissues without peritoneal carcinomatosis. **(D)** For pathways analysis of green subset, identification of pathways can be grouped in four clusters: epithelial–mesenchymal transition, receptors and metabolism, inflammation, and signaling. **(E)** Table showing the fold change of expression of the top 10 upregulated and downregulated genes included in green subset (fold change of <−2 or >+2 and p-value of < 0.05).

**Figure 3:**
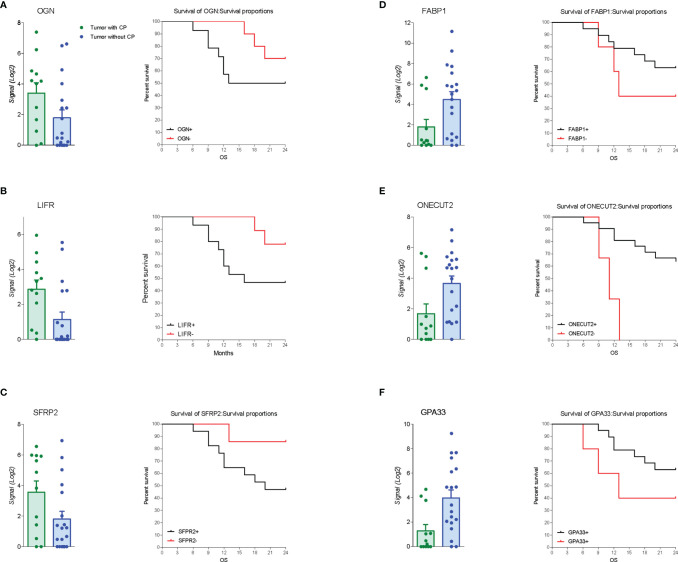
Gene expression and survival curve. Left panel: Relative mRNA expression levels extract from RNA-seq analysis and overall survival of patients according to up regulated genes expression of **(A)** OGN, **(B)** LIFR, and **(C)** SFRP2. Right panel: Relative mRNA expression levels extract from RNA-seq analysis and overall survival of patients according to down regulated genes expression of **(D)** Fabp1, **(E)** Onecut2, and **(F)** Gpa33.

The RNA transcription profile by AmpliSeq Transcriptome analysis (RNA-seq) of the two patient cohorts was carried out on paired samples of GCs and their matched normal tissues. The principal component analysis (PCA) of transcriptome shown in **Figure 2** highlighted the dissimilarities between GC samples obtained from patients with and without PC, showing only a partial overlap of the two groups. These results were confirmed by Venn diagram analysis of differentially expressed transcripts. As shown in **Figure 2**, this analysis allowed the identification of 341 transcripts belonging to AC+C subsets that were differentially modulated only in the cancer tissues with patients with peritoneal involvement. Specifically, 79 genes were upregulated and 262 downregulated (**Figure 2**). The *per-pathway* analysis of these differentially expressed genes using the TAC software (Affymetrix) demonstrated that the most modulated pathways in GC tumoral tissue belong to the EMT pathways, receptors and metabolism, inflammation, and signaling clusters (**Figure 2**). Analysis of differentially expressed (most upregulated and downregulated genes) in two cohorts of patients with GC (with or without PC) demonstrated that the top three upregulated genes were osteoglycin (Ong), LIFR, and secreted frizzled related protein 2 (Sfrp2); whereas the top three downregulated were fatty acid–binding protein 1 (Fabp1), one cut homeobox 2 transcriptional factor (Onecut2), and Ig superfamily protein glycoprotein A33 (Gpa33) genes (**Figure 2**). Whereas all six genes showed some degrees of correlation with patient survival (**Figure 3**), only the relative expression of Onecut2 and LIFR was statistically correlated with reduced patient survival at univariate analysis (P < 0.05). However, because, in comparison with normal mucosa, the expression of Onecut2 mRNA (39) was upregulated in the bulk tumor of patients with GC without peritoneal involvement but downregulated in those showing PC, we have focused our attention on LIFR.

### LIFR Expression Is Increased in Mucosa of Patient With GC With Peritoneal Carcinomatosis

**Figure 4:**
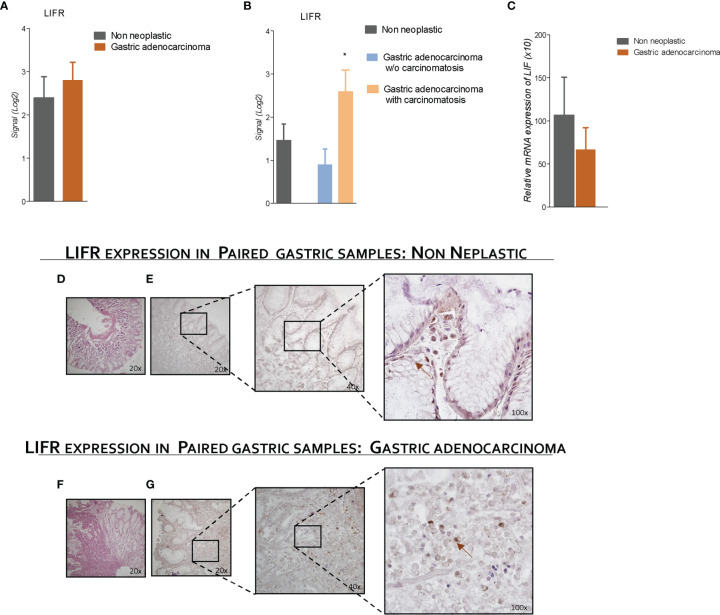
LIFR is a negative prognostic factor for survival of a patient with GC with carcinomatosis. The expression of LIFR and LIF was examined in surgical samples from non-neoplastic and gastric adenocarcinoma mucosa obtained by patients with GC underwent surgery for GC treatment. Data shown are follows: Gene expression of LIFR (Log2) **(A)** in non-neoplastic vs. neoplastic mucosa and **(B)** in non-neoplastic and gastric adenocarcinoma w/o carcinomatosis vs. adenocarcinoma with carcinomatosis. **(C)** Relative mRNA expression of LIF. **(D)** H&E staining of non-neoplastic mucosa (magnification, ×20). **(E)** IHC staining of non-neoplastic mucosa (magnification, ×20, ×40, and ×100). **(F)** (hematoxylin and eosin) H&E staining of gastric adenocarcinoma mucosa (magnification, ×20). **(G)** IHC staining of IHC staining of gastric adenocarcinoma mucosa (magnification, ×20, ×40, and ×100). * represents statistical significance versus Non neoplastic tissue.

To explore the role of LIFR and LIF in GC, we have then assessed LIFR expression in 31 tumor samples from patients with GC and compared them to the corresponding non-neoplastic mucosa. The results of this experiment demonstrated that LIFR expression in GC tissues was similar to that detected in paired samples of non-neoplastic mucosa (**Figure 4**). However, when patients with GC with or without peritoneal disease were compared, we found that LIFR expression was significantly increased in patients with PC (P-value of <0.05) (**Figure 4**). These findings were confirmed by LIFR IHC staining on GC biopsies. As shown in **Figure 4**, LIFR expression was detected as a faint signal in gastric glands on the normal mucosa, but the signal increased dramatically in the cancer tissues, showing a strong localization on the cell membrane of cancer cells (arrow), whereas some scattered signals were also detected in the tumor matrix (**Figure 4F**). Furthermore, to investigate the role of LIF/LIFR signaling, LIF mRNA expression level was assessed in paired samples of neoplastic and non-neoplastic mucosa of these patients, and, as shown in **Figure 4**, mRNA LIF expression showed a trend, although not significant, toward reduction in GC samples compared with non-neoplastic mucosa.

### LIF and LIFR Expression in GC Cell Lines

**Figure 5:**
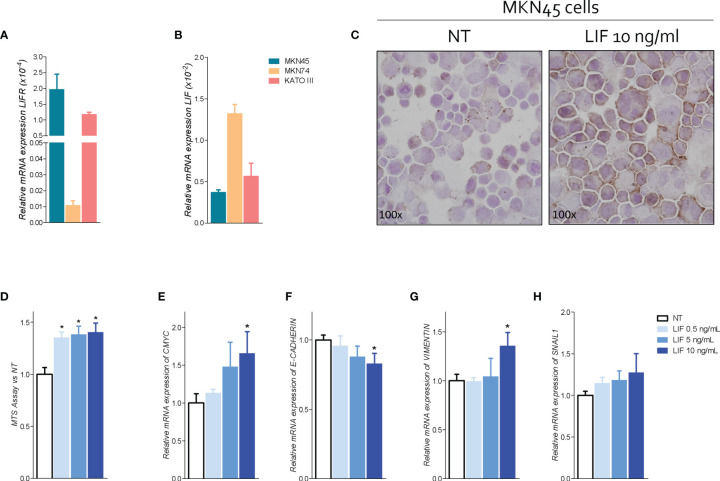
LIFR activation promotes cell proliferation and EMT in MKN45 cells. Relative mRNA expression **(A)** LIFR and **(B)** LIF in CG cell lines. **(C)** IHC staining of LIFR in MNK45 cell lines on the left untreated and on the right triggered with LIF (10 ng/ml; magnification, ×100). MKN45 cells were serum-starved and primed with LIF (0.5, 5, and 10 ng/ml). Data shown are as follows: **(D)** dose–response curve of LIF (0.5, 5, and 10 ng/ml) determined using MTS assay on MKN45 cells. Each value is expressed relative to those of non-treated (NT), which are arbitrarily settled to 1. Results are the mean ± SEM of 10 samples per group. Relative mRNA expression of **(E)** the proliferation marker C-Myc and EMT markers **(F)** E-cadherin, **(G)** vimentin, and **(H)** Snal-1. Each value is normalized to Gapdh and is expressed relative to those of positive controls, which are arbitrarily settled to 1. Results are the mean ± SEM of five samples per group (* represents statistical significance versus NT, and # versus LIF, p < 0.05).

Because the abovementioned data demonstrated that LIFR expression increases in patients with peritoneal involvement, we have then investigated whether the LIF/LIFR signaling drives the EMT transition using GC cell lines (**Figure 5A**) and found that the poorly differentiated cell line MKN45 shows the strongest expression of LIFR in comparison with KATO III and the more differentiated cell line, MKN74. In contrast, expression of LIF mRNA displayed an opposite trend, with MKN45 showing the lower expression and MKN74 showing the higher expression (**Figure 5A**), further confirming that LIF and LIFR were oppositely regulated, as observed in human samples (**Figure 4**) (36). Thus, we have used MKN45 cells in the following experiments.

To investigate the role of LIF/LIFR in modulating GC cells proliferation and function, MKN45 cells were cultured with increasing concentrations of LIF at 0.5, 5, 10, 50, and 100 ng/ml, and cell proliferation was assessed as detailed in Materials and Methods. Data shown in **Figure 5** demonstrated that exposure to LIF induced LIFR expression, as assessed by LIFR staining by ICC (**Figure 5**), and also promoted cell proliferation in a concentration-dependent manner as shown by results of MTS proliferation assay and relative mRNA expression of CMYC (**Figure 5**). Importantly, however, challenging MKN45 cells with higher concentrations of LIF at 50 and 100 ng/ml resulted in a growth-retardation effect, suggesting that, at these concentrations, LIF might be directly cytotoxic (**Figure S1**) (36).

Subsequently, we have investigated the effect of LIF on MKN45 cell cycle and apoptosis (**Figure S1**). LIF at the concentration of 10 ng/ml modulated cell proliferation and cycle, reducing the percentage of G0-G1 cells while increasing the percentage of MKN45 cells in in S-G2-M phases (**Figure S1**). Again, these effects were biphasic and higher concentrations of LIF (10 and 100 ng/ml) promoted a cell growth arrest (**Figure S1**). Thus, additional experiments were performed using LIF (10 ng/ml) as the maximal effective concentration.

Because LIFR promotes EMT in various cell systems, we have investigated the expression of E-cadherin, vimentin, and SNAIL1, the three well-recognized biomarkers of EMT, in MKN45 cells (40). The results of these experiments demonstrated that exposure of MKN45 to LIF (0.5, 5, 10, 50, and 100 ng/ml), for 48 h promoted a concentration-dependent reduction of E-cadherin mRNA expression (**Figure 5**), which was statistically significant (p < 0.05) at 10 ng/ml, while increasing the expression of vimentin and SNAIL1 mRNA in the same range of concentrations (**Figure 5G**). These effects were lost at higher concentrations, LIF at 50 mg/ml, due to increased apoptosis rate and cell growth arrest (**Figure S1**). Collectively, these data suggest that LIFR agonism promotes cells growth and EMT of MKN45 cells.

**Figure 6:**
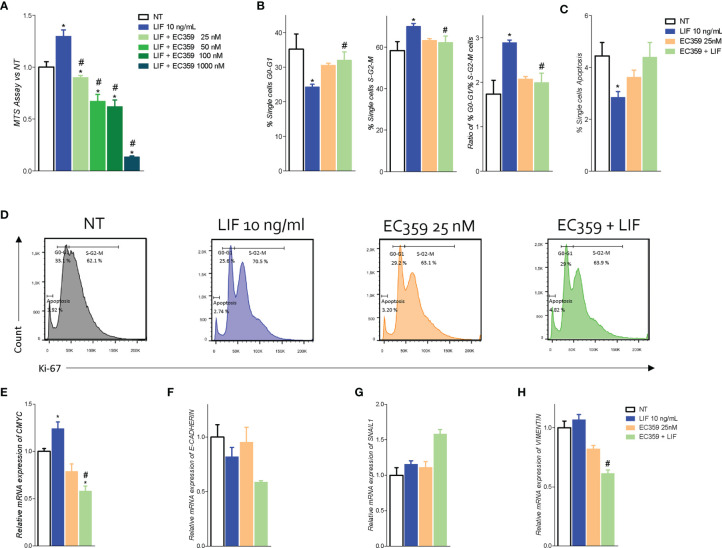
LIFR antagonist EC359 hinders cell cycle progression, increases apoptosis rate in MKN45 cells and inhibits EMT process. **(A)** Dose–response curve of EC359 (25, 50, 100, and 1,000 nM) determined using MTS assay on MKN45 cells (n = 10). MKN45 cells were serum-starved and triggered with LIF (10 ng/ml), EC359 (100 nM), and LIF + EC359 for 48 h. Cell cycle phase analysis was performed by Ki-67/DAPI staining through IC-FACS. Data shown are follows: percentage of **(B)** from left to right cell in G0-G1 cell cycle phases, S-G2-M cell cycle phases, and ratio between % G0-G1 and % S-G2-M. **(C)** Percentage of apoptotic cells. **(D)** Representative IC-FACS showed cell cycle fraction and apoptosis rate in NT, LIF (10 ng/ml), EC359 (25 nM), and LIF + EC359. Results are the mean ± SEM of three samples for group (* represents statistical significance versus NT, and # versus LIF, p < 0.05). Relative mRNA expression of **(E)** the proliferation marker C-Myc and EMT markers **(F)** E-Cadherin, **(G)** Snal-1, and **(H)** vimentin. Each value is normalized to Gapdh and is expressed relative to those of positive controls, which are arbitrarily settled to 1. Results are the mean ± SEM of five samples per group (* represents statistical significance versus NT, and # versus LIF, p < 0.05).

To further shed light in these findings to LIFR activation, we have then investigated whether LIFR inhibition effectively reversed this pattern. In these studies, we used EC359 as LIFR inhibitor. EC359 is a small molecule that selectively binds LIFR and downregulates its pro-oncogenic effects *in vitro* and *in vivo (* 31). For this purposes, MKN45 cells were growth in a medium with LIF (10 ng/ml), with or without increasing concentrations of EC359 at 25, 50, 100, and 1,000 nM, for 48 h. As shown in **Figure 6**, exposure to LIF again promoted cell proliferation as measured by MTS, and this effect was reversed in a concentration-dependent manner by EC359 (**Figure 6**). The above effects were statistically significant already at a concentration of 25 nM, whereas EC359 was cytotoxic at 1,000 nM. Similarly, the mRNA expression of CMYC was statistically reduced by 25 nM EC359 (**Figure 6**). In addition, the LIFR inhibition modulated the cell cycle as shown by Ki-67/DAPI IC-FACS staining (**Figure 6**). The cell cycle analysis revealed that EC359 alone did not decreased the rate of proliferative GC cells compared with untreated cells; instead, EC359, in combination with LIF, effectively reversed the effect of LIF in a statistically significant manner (p < 0.05), blocking the shift from resting cell in G0-G1 cell cycle phase to S-G2-M, as also demonstrated by the calculations of ratio between percent of G0-G1 and S-G2-M cells (**Figure 6**). Moreover, EC359 increased the apoptosis cell rates, which was diminished by LIF (**Figure 6**). Consistent with these findings, LIFR inhibition by EC359 reversed EMT features in MKN45 cells challenged with LIF. As shown in **Figure 6F**, at the concentration of 25 and 100 nM, EC359 downregulated E-cadherin and reduced the expression of vimentin. Taken together, these data demonstrate that EC359 effectively reverses GC cell proliferation and EMT promoted by LIF/LIFR signaling.

**Figure 7:**
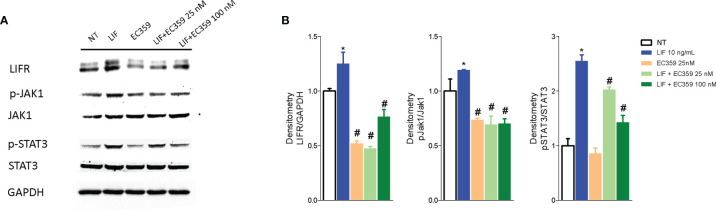
Analysis of JAK-STAT signaling pathway. Representative Western blot analysis of **(A)** LIFR, JAK1 and phospho-JAK1, STAT3 and phospho-STAT3, and proteins in MKN45 exposed to LIF (10 nM) alone or in combination with EC359 (25 nM and 100 nM) for 20 min. GAPDH was used as loading control. **(B)** Densitometric analysis demonstrating LIFR expression, phospho-JAK1/JAK1, and phospho-STAT3/STAT3 ratio. The blot shown is representative of another one showing the same pattern. (* represents statistical significance versus NT, and # versus LIF, p < 0.05).

Because LIF/LIFR activation promotes a downstream signaling that involves several kinases, we have then investigated whether challenging MKN45 cells with LIF promotes JAK and STAT3 phosphorylation. The results of these experiments demonstrated that LIF at the concentration of 10 ng/ml increases the expression of LIFR and promotes the phosphorylation of both JAK and STAT3 and that these effects were reversed by LIFR inhibition by EC359 at 100 nM (**Figures 7A, B**).

**Figure 8:**
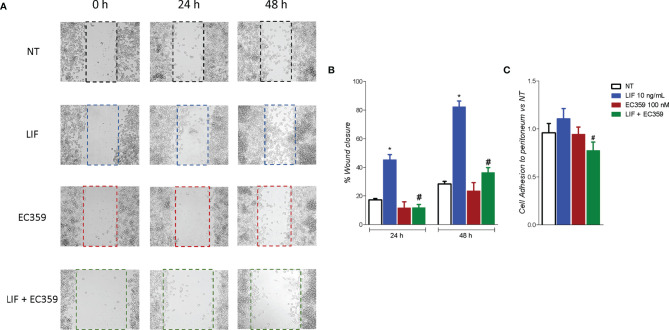
LIFR antagonism inhibits MKN45. **(A, B)** A scratch wound healing assay is shown. MKN45 cell monolayers were scraped in a straight line using a p200 pipette tip to create a “scratch”, and then, they are left untreated or primed with LIF (10 ng/ml), EC359 (100 nM), and LIF + EC359. The wound generated was imaged at 0, 24, and 48 h of incubation with the indicated compounds. **(A)** The images show cell migration at the three times point indicated. **(B)** Images obtained points were analysed measuring scraped area and its closure vs. the first time point at 0 h. Results are the mean ± SEM of three samples per group. **(C)** Cell adhesion to peritoneum. Experiment was conducted in quintuplicate (* represents statistical significance versus NT, and # versus LIF, p < 0.05).

To evaluated whether modulation of MKN45 by LIF promotes the acquisition of a migratory phenotype, we have performed a scratch wound healing assay, a validated method to functionally assess EMT (**Figure 8A**). For these purposes, MKN45 cells were growth in a complete medium, and, on the day 0, after a scratch was produced as described in Material and Methods, cells were challenged with LIF (10 ng/ml) and EC359 (100 nM) or the combinations of the two. Cell migration was assessed by measuring the area between the two scratch margins at different time points: 0, 24, and 48 h. As illustrated in **Figure 8**, exposure to LIF promoted cell migration and wound closure with a reduction of the wound area of 45.41% at 24 h and 82.23% at 48 h. This pattern was reversed by exposure to EC359 (p < 0.05). In addition, EC359 alone reduced the percentage of wound closure compared with untreated cells, but these changes were not statistically significant. Similar findings were observed assessing the adhesion of MKN45 cells to the peritoneum. In this assay, although LIF promoted MKN45 adhesion to the mouse peritoneum, the effect was again significantly attenuated by co-treating the cells with EC359 by ≈30% (**Figure 8**). In summary, these results demonstrate that LIFR inhibition decreases LIF-induced ability to gain the migratory phenotype of MKN45 cells.

## Discussion

The LIF/LIFR signaling has been identified as a potential therapeutic target in the treatment of several cancers. In the present study, we report the transcriptome profiling (RNA-seq) of a group of patients with GC with or without PC. This investigation allowed the identification of LIFR as one of the highest expressed genes in patients with peritoneal involvement and as a strong predictor of a poor prognosis in these patients. In addition, we have shown that activation of LIF/LIFR signaling in GC cells promotes the acquisition of a mesenchymal phenotype, suggesting a potential mechanistic role of LIF/LIFR signaling in the development of peritoneal metastasis.

The PC is a relatively common localization of metastasis in GC, occurring in up to 14% of newly diagnosed patients with GC, and is the most common site (~50%) of recurrence in patients with GC after radical surgery (41–43). In our series, as expected, presence of peritoneal metastasis was a strong predictor of poor prognosis, and mean survival time of patients with positive peritoneal cytology or macroscopic evidence of peritoneal metastasis at surgery (Cy+/P+) was approximately 12 months, significantly lower in comparison with the 60 months median survival observed in patients that were Cy0/T0. These data are in agreement with previous findings, confirming the fact that the development of a peritoneal disease is a strong predictor of a shorter-term survival in patients with GC.

In addition to the presence of peritoneal disease, by the transcriptome analysis of paired samples of neoplastic tissue and normal gastric mucosa, we have identified a group of six differentially expressed genes that predict poor prognosis: ONG, FABP1, LIF, ONECUT2, SFRP2, and GPA33. More specifically, we have shown that the top three upregulated genes—ONG, LIFR, and SFRP2, and the top three downregulated genes—FABP1, ONECUT2, and GPA33, were all associated with a poor prognosis, although statistically significant difference was detected only for the expression of ONECUT2 and LIFR (P < 0.05).

ONECUT2 belongs to the family of the ONECUT transcription factors, a small group of evolutionarily conserved proteins that play a role in the embryo, liver, pancreas, and neuronal system development (44). Although a role for ONECUT2 in cancer is not well defined, there is evidence that the expression of this gene is aberrantly upregulated in a variety of cancers including hepatocellular carcinoma, prostate cancer, colorectal cancer, and ovarian cancer, suggesting a role for this transcription factor in the modulation of cancer progression (45). Despite the fact that, similar to a previous study, we have found that ONECUT2 gene expression was increased in the neoplastic tissues in comparison with paired samples obtained from non-neoplastic mucosa, we have found that reduced levels of ONECUT2, rather than its induction, are a poor prognosis predictor in patients with GC and peritoneal disease (39). The reason for this discrepancy is unclear, because overexpression of ONECUT2 in MKN54 and AGS, two GC cell lines, promotes cell proliferation and migration. However, others have reported that ONECUT2 regulation occurs through epigenetic regulation and hypomethylation of that CpGs in the promoter of ONECUT2, and this regulation occurs primarily in promoting intestinal differentiation of gastric mucosa. Accordingly, it has suggested that tissues levels of ONECUT2, gene and protein, might have utility in detecting intestinal metaplasia and might represent a biomarker of initial stages of gastric carcinogenesis. In contrast, the role of ONECUT2 in advanced disease is less defined (46).

The formation of peritoneal metastasis in GC is a multistep process, whereby cancer cells detach from primary tumor, migrate and attach to distant peritoneum, followed by invasion into sub-peritoneal tissues and cell proliferation to form detectable metastasis (47). Despite the clinical relevance, the specific molecular mechanisms that drive the formation of peritoneal metastasis in GC remain poorly understood, although previous studies using paired samples of primary and metastatic tumors have identified several putative mediators, mostly related to EMT remodeling, cell motility, and cytoskeleton rearrangement (48). Here, we report that the development of peritoneal disease in our series of patients with GC is associated with a robust upregulation of the LIFR in the primary tumors. This finding prompted us to further investigate whether the LIF/LIFR system was involved in promoting the EMT phenotype, a process that involves a deep reprogramming of the cancer cells genes. LIFR is an heterodimeric membrane receptor complex composed by LIFRβ and GP130 (49, 50), and although the receptor lacks an intrinsic tyrosine kinase activity, LIFR/GP130 complex constitutively associates with JAK-Tyk family of cytoplasmic tyrosine kinases, which facilitates downstream signaling and STAT3 phosphorylation activation. Several tumors exhibit upregulated JAK/STAT, ERK/MAPK, and PI3K/AKT signaling *via* autocrine or paracrine activation of LIF/LIFR GP130, and this pathway significantly contributes to EMT in several cancers, disease progression, and a poorer relapse-free survival in several cancers (51). LIF also participates to cross-talk between tumor cells and matrix fibroblasts to mediate the pro-invasive activation of stromal fibroblasts (52) and promotes drug resistance to HDAC inhibitors (53). By using GC cell lines, we have shown that LIFR expression varies from one line to another and that MKN45 cells were the cells with the highest expression. Challenging these cells with LIF promotes the acquisition of migratory phenotype, and this is associated with the acquisition of molecular signature of EMT and these changes are associated with LIF/LIFR signaling as assessed by measuring JAK and STAT3 phosphorylation. Of relevance, the LIFR inhibitor, EC359 (IC_50_ values of 10.2 nM), reversed these changed. Cotreating MKN45 cells with EC359 also reversed JAK and STAT3 phosphorylation induced by exposure of MKN45 cells to LIF, as well as regulation of E-cadherin and vimentin in a concentration-dependent manner, further confirming the role of LIF/LIFR in promoting EMT as well as acquisition of migratory phenotype of GC cells.

Cytoskeletal remodeling is closely related with tumor migration, invasion, and metastasis (54). LIFR plays an essential role in regulation of actin filament dynamics by modulating the expression of vimentin. Consistent with this background, we demonstrate that pharmacological inhibition of LIFR negatively regulates the expression of vimentin and that this effects are associated with a reduce cell motility and impaired migration (55, 56). Vimentin plays an important role in tumor invasion and metastasis (57), and its counter-regulation is a further evidence of the role that LIF/LIFR signaling plays in the modulation of EMT process. Of relevance, LIFR activation positively regulates vimentin expression and downregulates E-cadherin *via* JAK and STAT3 phosphorylation, and LIFR antagonism reversed this pathway (55–57).

In conclusion, by NGS RNA-seq analysis, we have identified LIF/LIFR pathway as an important mechanism in disease progression in GC. High levels of expression of LIFR mRNA in tumor tissues predict poor prognosis and reduced response to therapy. In addition, by using GC cell lines, we have shown that LIFR activation results in JAK STAT3 phosphorylation and EMT as demonstrated by vimentin induction and blunted expression of E-cadherin. These molecular changes are associated with a migratory phenotype of GC cell lines and are reversed by LIF/LIFR antagonism. Together, we suggest that targeting LIF/LIFR signaling might have utility in management of GC.

## Data Availability Statement

The datasets presented in this study can be found in online repositories. The names of the repository/repositories and accession number(s) can be found at https://data.mendeley.com/drafts/9t86hd78sj, 10.17632/9t86hd78sj.1.

## Ethics Statement

The studies involving human participants were reviewed and approved by FI00001 no. 2266/2014, granted on February 19, 2014, and by FIO0003 no. 36348 granted on May 6, 2020. The patients/participants provided their written informed consent to participate in this study.

## Author Contributions

SF, LG, and AD contributed to conception and design of the study. AZ and SF provided research funding. LG, EM, and AD provided human samples. CD, SM, EM, and MB performed the data analysis. CD, SM, and EM performed the statistical analysis. SF, CD, SM, EM, and ED wrote the manuscript. CD, SM, RR, MB, RB, and GU contributed experimental settings. All authors contributed to the article and approved the submitted version.

## Funding

This work was partially supported by grant from the Italian MIUR/PRIN 2017 (2017FJZZRC).

## Conflict of Interest

The authors declare that the research was conducted in the absence of any commercial or financial relationships that could be construed as a potential conflict of interest.

## Publisher’s Note

All claims expressed in this article are solely those of the authors and do not necessarily represent those of their affiliated organizations, or those of the publisher, the editors and the reviewers. Any product that may be evaluated in this article, or claim that may be made by its manufacturer, is not guaranteed or endorsed by the publisher.

## Supplementary Material

Supplementary Figure 1 MKN45 cells were serum-starved and left untreated or primed with LIF (10,50,100 ng/ml). Data shown are: **(A)** MTS assay. Each value is expressed relative to those of non-treated (NT), which are arbitrarily settled to 1. Results are the mean ± SEM of 10 samples per group. Relative mRNA expression of **(B)** the proliferation marker C-myc and EMT markers E-cadherin, Snal-1, and vimentin. Each value is normalized to Gapdh and is expressed relative to those of NT, which are arbitrarily settled to 1. Results are the mean ± SEM of five samples per group (* represents statistical significance versus NT, and # versus LIF, p < 0.05). Cell cycle phase analysis were performed by Ki-67/DAPI staining through IC-FACS analysis. Data shown are: percentage of **(D)** Representative IC-FACS showed cell cycle fraction and apoptosis rate in NT, LIF (10 ng/ml), EC359 (25 nM), and LIF + EC359. **(E)** cell in G0-G1 cell cycle phases, S-G2-M cell cycle phases, and ratio between % G0-G1 and % S-G2-M. **(F)** Percentage of Apoptotic cells. Results are the mean ± SEM of three samples per group (* represents statistical significance versus NT, and # versus LIF, p < 0.05).
