# Supplementary figures and images for: Next-Generation Sequencing Analysis of Gastric Cancer Identifies the Leukemia Inhibitory Factor Receptor as a Driving Factor in Gastric Cancer Progression and as a Predictor of Poor Prognosis
Source: Front Oncol. 2022 Jun 30;12:939969. doi: 10.3389/fonc.2022.939969 (PMC9280277; doi:10.3389/fonc.2022.939969)

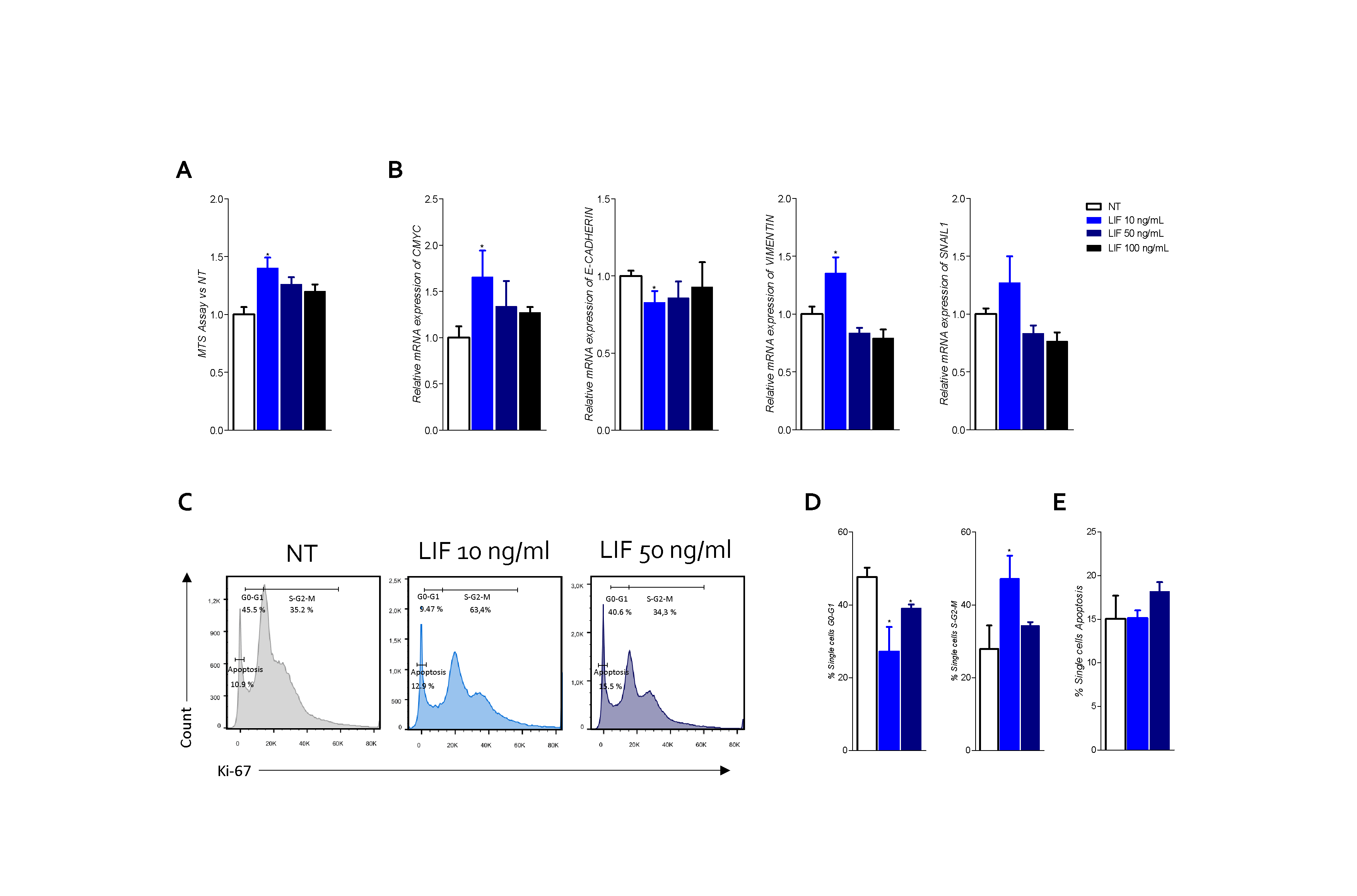

Supplement: Supplementary Figure 1 — MKN45 cells were serum-starved and left untreated or primed with LIF (10,50,100 ng/ml). Data shown are: (A) MTS assay. Each value is expressed relative to those of non-treated (NT), which are arbitrarily settled to 1. Results are the mean ± SEM of 10 samples per group. Relative mRNA expression of (B) the proliferation marker C-myc and EMT markers E-cadherin, Snal-1, and vimentin. Each value is normalized to Gapdh and is expressed relative to those of NT, which are arbitrarily settled to 1. Results are the mean ± SEM of five samples per group (* represents statistical significance versus NT, and # versus LIF, p < 0.05). Cell cycle phase analysis were performed by Ki-67/DAPI staining through IC-FACS analysis. Data shown are: percentage of (D) Representative IC-FACS showed cell cycle fraction and apoptosis rate in NT, LIF (10 ng/ml), EC359 (25 nM), and LIF + EC359. (E) cell in G0-G1 cell cycle phases, S-G2-M cell cycle phases, and ratio between % G0-G1 and % S-G2-M. (F) Percentage of Apoptotic cells. Results are the mean ± SEM of three samples per group (* represents statistical significance versus NT, and # versus LIF, p < 0.05). [file Image_1.tif]
